# Epidemiology and genetic diversity of circulating dengue viruses in Medellin, Colombia: a fever surveillance study

**DOI:** 10.1186/s12879-020-05172-7

**Published:** 2020-07-02

**Authors:** Jacqueline Kyungah Lim, Mabel Carabali, Erwin Camacho, Diana Carolina Velez, Andrea Trujillo, Jorge Egurrola, Kang-Sung Lee, Ivan Dario Velez, Jorge E. Osorio

**Affiliations:** 1 Dengue Vaccine Initiative, International Vaccine Institute, SNU Research Park, 1 Gwanak-ro, Gwanak-gu, Seoul, 08826 Republic of Korea; 2 Department of Epidemiology, Biostatistics and Occupational Health, McGill University, 845 Sherbrooke St., W, Montreal, Quebec, H3A 0G4 Canada; 3 Investigaciones Biomedicas, Universidad de Sucre, Cra 28 # 5-267, Barrio Puerta Roja, Sincelejo, Sucre Colombia; 4 Programa de Estudio y Control de Enfermedades Tropicales (PECET), Universidad de Antioquia, calle 67 No. 53, 108 Medellín, Antioquia Colombia; 5 Department of Pathobiological Sciences, University of Wisconsin, 500 Lincoln Dr, Madison, WI 53706 USA

**Keywords:** Dengue, Colombia, Surveillance, Genotyping

## Abstract

**Background:**

Dengue fever is a major public health problem in Colombia. A fever surveillance study was conducted for evaluation of the clinical, epidemiological, and molecular patterns of dengue, prior to Chikungunya and Zika epidemics.

**Methods:**

In November 2011–February 2014, a passive facility-based surveillance was implemented in Santa Cruz Hospital, Medellin, and enrolled eligible febrile patients between 1 and 65 years-of-age. Acute and convalescent blood samples were collected 10–21 days apart and tested for dengue using IgM/IgG ELISA. RNA was extracted for serotyping using RT-PCR on acute samples and genotyping was performed by sequencing.

**Results:**

Among 537 febrile patients enrolled during the study period, 29% (*n* = 155) were identified to be dengue-positive. Only 7% of dengue cases were hospitalized, but dengue-positive patients were 2.6 times more likely to be hospitalized, compared to non-dengue cases, based on a logistic regression. From those tested with RT-PCR (*n* = 173), 17 were dengue-confirmed based on PCR and/or virus isolation showing mostly DENV-3 (*n* = 9) and DENV-4 (*n* = 7) with 1 DENV-1. Genotyping results showed that: DENV-1 isolate belongs to the genotype V or American/African genotype; DENV-3 isolates belong to genotype III; and DENV-4 isolates belong to the II genotype and specifically to the IIb sub-genotype or linage.

**Conclusions:**

Our surveillance documented considerable dengue burden in Santa Cruz comuna during non-epidemic years, and genetic diversity of circulating DENV isolates, captured prior to Chikungunya epidemic in 2014 and Zika epidemic in 2015. Our study findings underscore the need for continued surveillance and monitoring of dengue and other arboviruses and serve as epidemiological and molecular evidence base for future studies to assess changes in DENV transmission in Medellin, given emerging and re-emerging arboviral diseases in the region.

## Background

Dengue infection, caused by dengue viruses (DENV 1–4) and transmitted by *Aedes* mosquitoes, is a major public health problem in tropical and sub-tropical countries, including Colombia [1]. Clinical presentations of dengue can range from dengue fever (DF); high fever, rash, and muscle and joint pain to severe dengue with plasma leakage, bleeding, or organ failure [2–4]. DF and severe dengue are major causes of mortality and morbidity with: 390 million DENV infections; 500,000 of severe dengue cases requiring hospitalization; and approximately 20,000 deaths estimated annually worldwide [2, 4].

An effective and safe vaccine against dengue is needed. Recently, the first dengue vaccine (Dengvaxia®, by Sanofi Pasteur) was licensed in multiple countries in Asia and Latin America. However, this vaccine has variable efficacy and has a restricted indication in dengue-exposed subjects only from 9 years and above, due to increased risk of severe dengue in seronegative subjects [4, 5].

In Colombia, dengue is hyper-endemic with circulation of all four serotypes, and there has been a significant increase in the number of cases of DF/severe dengue in the last 10 years, with epidemics occurring every 3–4 years [1, 6]. Colombia experienced an outbreak in 2016 with 103,822 dengue cases reported [7] since a peak observed in 2013 with 65,464 lab-confirmed cases among 127,000 clinical cases [8].

There is a well-established national dengue surveillance system. However, most of existing data are focused on hospitalized cases, even though outpatient dengue accounts for the greatest burden of disease, and data on dengue among adults are relatively scarce compared to what is available for children [1, 9]. To understand epidemiology and genetic diversity of circulating DENVs, a health facility-based fever surveillance was launched in a catchment area population of approximately 100,000 residents in Medellin, Colombia.

## Methods

### Study site and population

The metropolitan area of Medellin in the State of Antioquia is the second largest city in Colombia. Dengue is endemic in Medellin with a reported annual incidence of 161–745/100,000 and a recent epidemic in 2016 [7]. With all 4 serotypes in circulation in Antioquia, the prevalent serotypes were DENV-2 and DENV-1 in 2000–2010 [6].

Population size for the catchment area was determined using an adjusted incidence of dengue, augmented to account for the level of under-reporting as previously documented in the region [10]. The overall sample size of the catchment area population was 45,193 and, considering for 20% loss follow-up, the population size of the catchment area was 54,232.

**Fig. 1:**
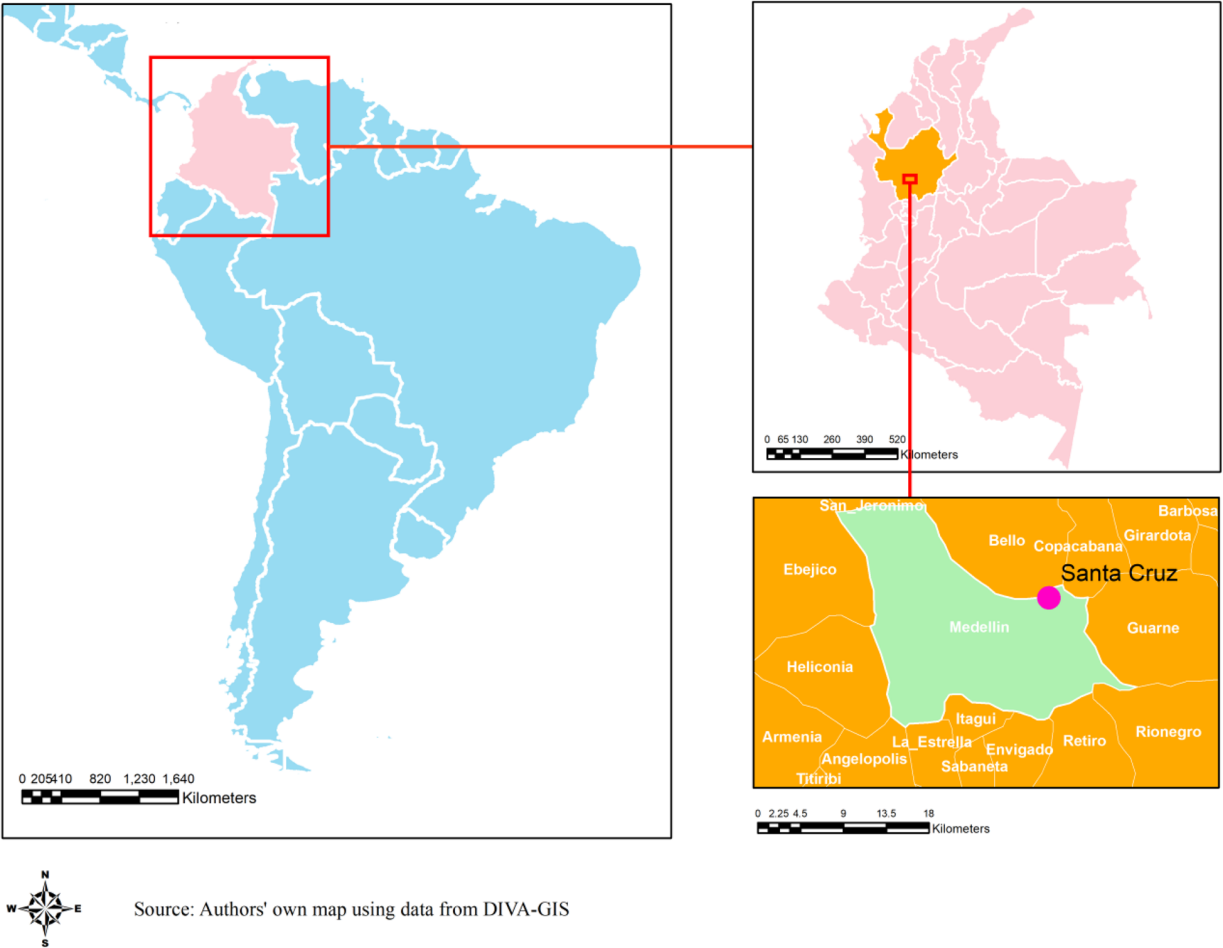
A map of the study area in Santa Cruz, Medellin, Colombia. The map shows the study area in Santa Cruz, Medellin, Colombia.

Santa Cruz comuna, one of the 16 sub-districts in Medellin with a population size of 107,869, was selected to be the catchment area for the surveillance. Approximately, 87% of the population are Mestizo and White, with 12% Afro-Colombians. In terms of socio-economic level, approximately 96% of the households belong to the socioeconomic stratum 2 (low). In Santa Cruz comuna, there are three basic health centers and Santa Cruz Hospital (SCH), a 48-bed medium-sized secondary care facility (Fig. 1).

### Study design

In the passive fever surveillance implemented at SCH, eligible subjects among febrile outpatients and hospitalized patients were enrolled and tested for dengue. Eligibility for dengue screening was based on the age (1–65 years), presence of fever (body temperature ≥ 37.5 °C) or history of fever for ≤7 days of duration, without localizing signs (i.e. fever caused by a localized infection or with other known/confirmed etiology), being resident of the Santa Cruz comuna, and not being a participant in any dengue vaccine clinical trials during the study period. Exclusion of infants < 1 year-of-age was based on consideration of the presence of maternal antibodies and challenges of infantile bleeding.

**Fig. 2:**
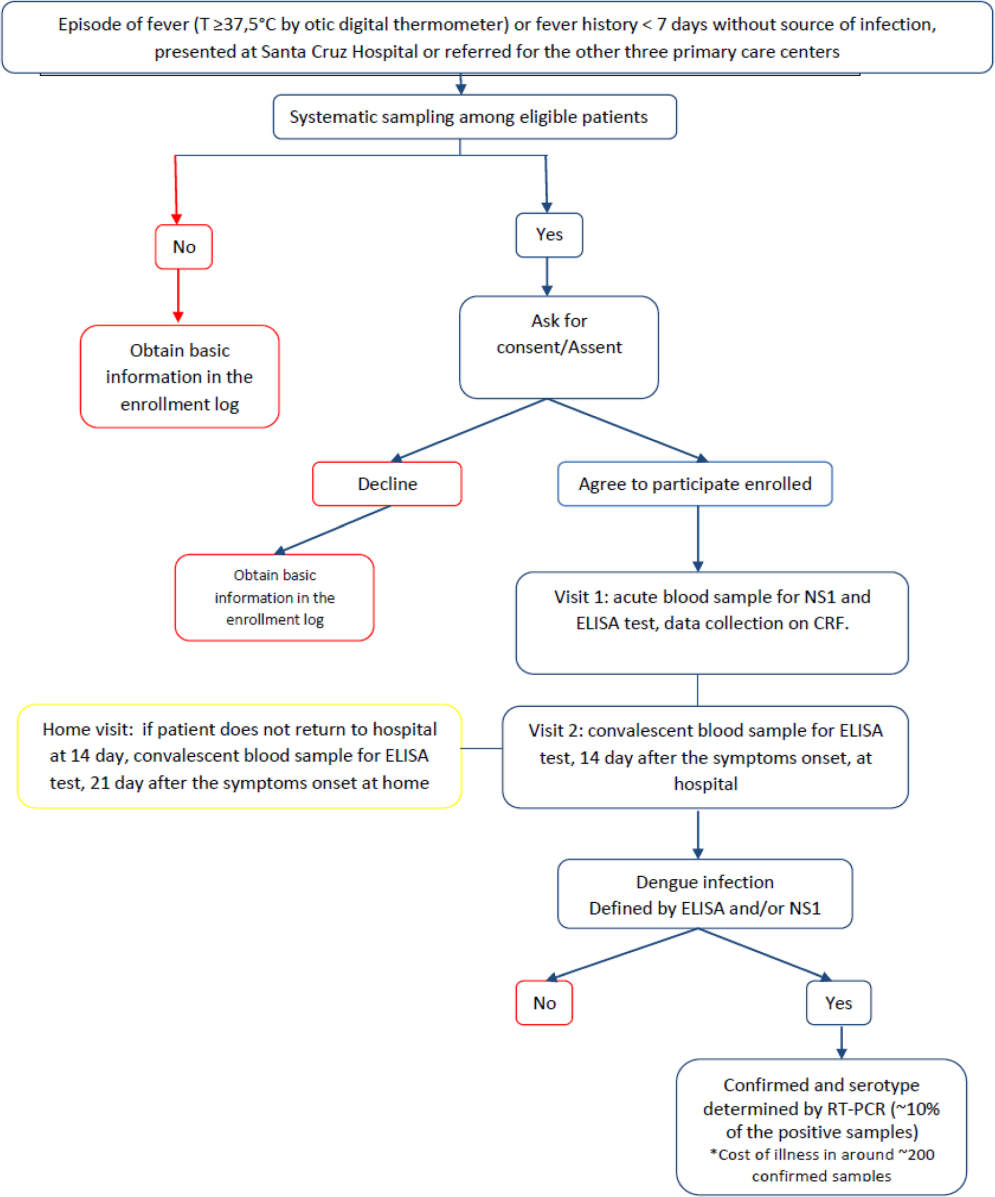
Patient flow for the passive fever surveillance at Santa Cruz Hospital. The chart shows the study flow when a febrile patient presents at Santa Cruz Hospital from screening and enrollment to lab testing

If eligible and agree to participate, the patient was referred to the study physician (Fig. 2). After collection of an acute sample of blood of 7–10 mL at enrollment, a study physician/nurse completed the surveillance case report form based on physical examination, to collect medical history and laboratory results. Then, a follow-up visit took place at SCH for collection of a convalescent blood sample and clinical data, between 10 and 14 days after visit 1. If the patient was not able to visit the hospital, a home visit was made within 21 days after visit 1.

### Laboratory analyses

At enrollment, subjects were tested for the detection of DENV NS1 antigen by rapid test [Standard Diagnostics (SD), Yongin-Si, Korea]. The RDT result was used as an initial screening tool for further testing. Blood samples were tested using dengue IgM/IgG ELISA (SD Dengue IgM & IgG Capture ELISA®, Standard Diagnostics, Yongin-Si, Korea) in Programa de Estudio y Control de Enfermedades Tropicales (PECET) in Universidad de Antioquia. Samples showing a test result ≥ the defined cut-off were considered to have presence of detectable anti-dengue IgM and IgG antibodies, which were interpreted as primary or recent/past infection.

Additionally, those acute serum samples which met at least one of the following eligibility criteria, underwent further molecular analyses at PECET and University of Wisconsin, Madison: (i) NS1 test positive on RDT in acute phase, (ii) IgM anti-DENV positive in acute and/or convalescent phase, and (iii) IgG anti-DENV positive in acute phase. Also, RT-PCR was performed on a small number of acute sera of the samples that stayed sero-negative between acute and convalescence phase on IgM capture ELISA.

The samples with negative results on RT-PCR and sero-negative results on paired IgM and IgG ELISA results were classified as non-dengue. A positive IgM or IgG serology in a single serum collected after day 5 of symptoms onset were diagnostic criteria of probable dengue [11]. Sero-conversion of anti-dengue IgM from negative in the acute phase to positive in the convalescent phase and/or virus detection (RT-PCR) in the acute serum specimen were considered laboratory-confirmed dengue. Confirmed- and probable-dengue were grouped together as dengue-positive cases in this analysis. Sero-conversion on IgM ELISA with sero-positive results on IgG ELISA was classified as secondary dengue infections. When the test shows sero-conversion of anti-dengue IgG from negative in the acute phase to positive in the convalescent phase, it was considered as either current or recent exposure with DENV.

### Virus isolation and serotyping

Viral isolation assays were conducted by inoculating the positive serum samples, in addition to a subset of samples which tested negative by RT-PCR but NS1 positive on RDT, into C6/36 cells monolayers. After 10 days of inoculation, supernatants were collected for RT-PCR tests and unattached cells were fixed for Immunofluorescence assays. For virus identification in the supernatants, RNA extractions (Zymo Viral RNA Kit – Zymo Research), Reverse Transcriptions (Superscript III First Strand cDNA Synthesis kit - Invitrogen), and multiplex PCR (One Taq 2X Master Mix – New England Biolabs), were conducted using serotype-specific primers described previously [12, 13]. Due to low band intensity or negative results obtained with these procedures, a second passage on C6/36 cells monolayers was performed. Supernatants were harvested after 7 days of inoculation and underwent viral molecular detection and typing.

### Envelope gene amplification and sequencing

Two overlapping fragments corresponding to the 5’UTR- NS2B region of DENV genome were amplified. Serotype-specific primers were used to synthetize cDNA (Superscript III First Strand cDNA Synthesis kit – Invitrogen) that was used as a template in the amplification of the two fragments (Q5 High Fidelity DNA Polymerase – New England Biolabs) with serotype-specific primers [14]. The primers that were used to amplify the two overlapping fragments along with additional internal primers, that hybridize in different regions of the target gene, were used to run individual sequencing reactions. Twelve sequence fragments were obtained for each isolate, after edition and selection of the best resolved regions in the received chromatograms. These fragments were assembled, with the ContigExpress Tool within the Vector NTI software (Invitrogen, Carlsbad, California, United States), and carefully reviewed to obtain sequences that contain the complete DENV envelope gene. Total sequence lengths varied by serotype; 1959 bp for DENV-1 isolate, 2653–2347 bp for DENV-3 isolates, and 1963–1077 bp for DENV-4 isolates.

### Variable construction and statistical analysis

First, a descriptive summary of characteristics is presented between dengue-positive (combining confirmed- and probable-dengue, following the WHO diagnostic criteria) and non-dengue patients [11]. Clinical diagnosis at admission, prior to lab-confirmation, was grouped as suspected dengue, undifferentiated fever, and non-dengue. Dichotomous variable was created for yellow fever vaccination history, comparing those self-reported to have been vaccinated vs. those who did not self-report vaccination or did not remember.

Categorical pair-wise comparisons were made between dengue-positive and non-dengue cases using Chi-square (*χ*2) and Fisher’s exact tests, with significance at *p*-value < 0.05. Comparison of continuous variables was performed using the student’s t-test and ANOVA. To identify characteristics associated with dengue positivity, independent variables, such as treatment type, and fever duration prior to visit, were investigated in univariable associations. Associations were expressed in terms of odds ratios (ORs) with 95% confidence intervals (CIs). All analyses were performed using SAS® version 9.4 (SAS Institute, Cary, North Carolina).

### Phylogenetic analysis

Full-length envelope gene sequence databases were constructed for each detected DENV serotype with available sequences in GenBank with known location and sampling date (last accessed in November 2018), using the NCBI Mass Sequence Downloader software [15]. Datasets were downsized by removing identical and highly similar (> 99.8%) sequences from the same year and country by clustering with the CD-HIT program [16, 17]. Resulting datasets (953 sequences for DENV-1, 545 for DENV-3 and 862 for DENV-4) were combined with the sequences in this study and aligned with MAFFT v7.402 software [18]. To identify the corresponding genotypes of the isolates, an initial phylogenetic analysis was performed by maximum likelihood (ML) reconstruction with ultrafast bootstrap approximation (UFBoot) using the IQ-TREE web server [19, 20].

To focus on the identified genotypes, sequences from the same year and country were down sampled when they were included in monophyletic clades to reduce overrepresentation. These filtered datasets (117 sequences for DENV-1, 123 for DENV-3, and 124 for DENV-4) were used to perform a ML analysis, to test the temporal structure in the data using TempEST v1.5.1 software [21]. With each alignment, a statistical selection of the best fit models of nucleotide substitution was performed with jModelTest software [22, 23]. Prior to molecular clock phylogenetics, the best-fit demographic model (constant-size population, exponential growth population, Bayesian Skyline and Bayesian SkyGrid priors) and the best fit clock model (strict clock and uncorrelated relaxed clock with log-normal distribution) were selected by estimation of marginal likelihood via path-sampling (PS) and stepping-stone sampling (SS) on a 1 million chain sampled for 100 path steps. Phylogenetic reconstructions were performed using BEAST v1.8.4 software [24] with a 100 million generations Markov Chain Monte Carlo (MCMC). Estimation of substitution rates and time of most recent common ancestor (TMRCA) for specific clades were performed using Tracer v1.7 [25]. Maximum clade credibility trees (MCC) were obtained with TreeAnotator v1.8.1 and 10% of the initial MCMC samples were discarded as burn-in.

### Ethical considerations

A written informed consent from (ICF) was obtained from each participant. For subjects 7 years old or younger, an informed consent was obtained from at least one parent or legal guardian. For those aged between 8 and 18 years, an assent form was obtained, plus informed consent from at least one parent or legal guardian. The protocol obtained ethical approvals from the Ethics Committee of the Universidad de Antioquia, Secretaria de Salud de Medellin, Metrosalud E.S.E/ Unidad Hospitalaria Santa Cruz and the Institutional Review Board (IRB) of International Vaccine Institute (IVI).

## Results

**Fig. 3:**
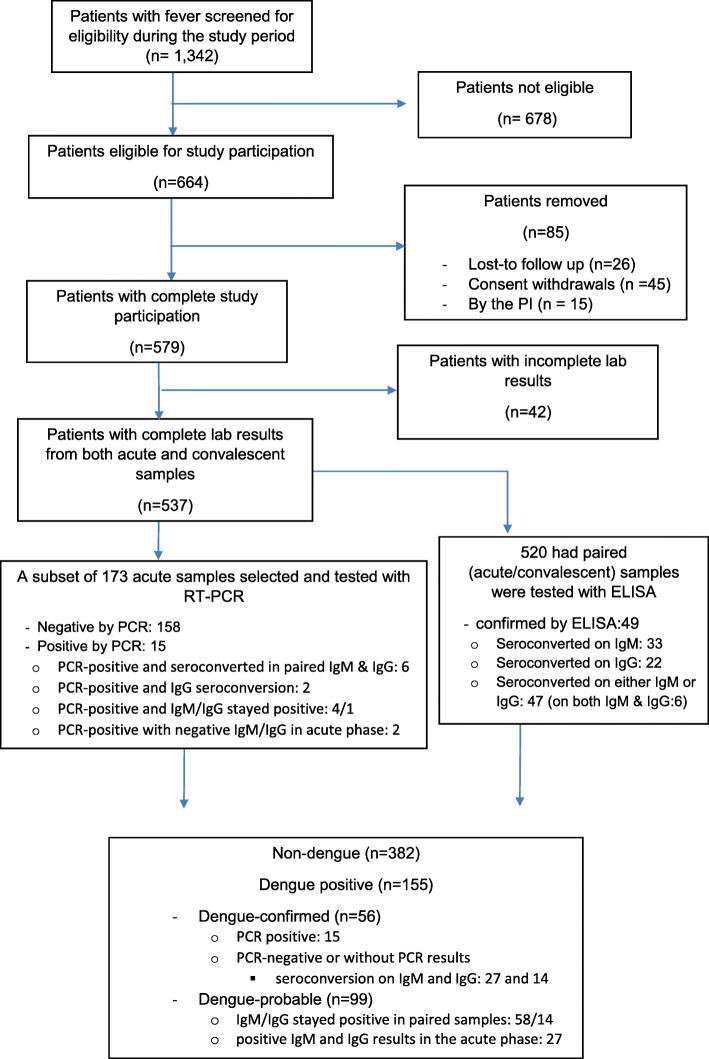
Flow chart describing the ascertainment of the febrile patients during the study period of November 2011–February 2014. The diagram shows how we reached the study population and the test results from collected samples, within the surveillance.

Among 1342 febrile patients screened for study participation during the 28-month study period, 664 patients were eligible and 579 patients agreed to participate in the study (Fig. 3). After removing 42 of them due to incomplete laboratory and clinical data, the analysis sample included 537 febrile subjects.

### General characteristics

**Fig. 4:**
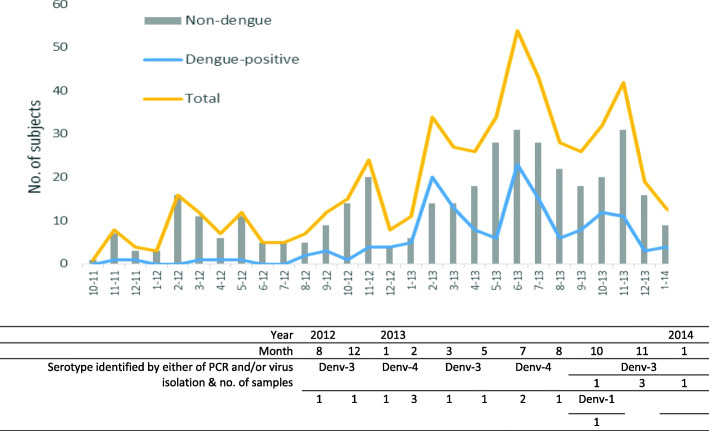
Monthly distribution of the enrolled febrile patients (*n* = 537) and dengue-positive patients (*n* = 155) with the serotype distribution in PCR-positive cases. The figure has two parts: the upper part shows monthly distribution of dengue-positive and non-dengue cases among the enrolled patients; and the lower part shows distribution of serotypes

Among 537 subjects, 29% (*n* = 155) were found to be dengue-positive patients, composed of 56 dengue-confirmed and 99 dengue-probable cases (Table 1). Confirmed- and probable-dengue were similar in terms of age, outcome of illness (hospitalization), and symptoms. In terms of monthly distribution of dengue-positive cases, there were peaks of dengue transmission, in January–March and in May–August (Fig. 4). These were followed by the known rainy seasons in March–May and September–December.

**Table 1:** Demographic and clinical characteristics of dengue-positive patients vs. non-dengue patients from the hospital-based fever surveillance established in Medellin, Colombia in 2012–2014

Characteristics	Dengue-positive (*n* = 155)	Non-dengue (*n* = 382)	*p*-value	Total (*N* = 537)
Age group (years)			0.286	
1–4	33 (21.3)	87 (22.8)		120 (22.4)
5–9	26 (16.8)	73 (19.1)		99 (18.4)
10–24	42 (27.1)	100 (26.2)		142 (26.4)
25–44	31 (20.0)	63 (16.5)		94 (17.5)
45–65	23 (14.8)	59 (15.5)		82 (15.3)
Female (%)	89 (57.4)	224 (58.6)	0.795	313 (58.3)
Mean days of fever since onset (SD)	3.72 (1.75)	3.35 (1.57)	**0.017**	3.45 (1.63)
Mean overall fever duration (SD)	4.85 (2.39)	4.48 (2.37)	0.111	4.59 (2.38)
Current fever at presentation	38 (24.5)	111 (29.1)	0.287	149 (27.8)
Temperature at enrollment			0.287	
Below 38°c	117 (75.5)	271 (70.9)		388 (72.3)
≥ 38°c	38 (24.5)	111 (29.1)		149 (27.8)
Fever duration prior to visit			**0.001**	
1–2 days	47 (30.3)	114 (29.8)		161 (30.0)
3–4 days	52 (33.6)	183 (47.9)		235 (43.8)
5–7 days	56 (36.1)	85 (22.3)		141 (26.3)
Prev. dengue infection (%)	5 (3.2)	15 (3.9)	0.698	20 (3.7)
YF vaccination (%)	55 (35.5)	183 (47.9)	**0.009**	238 (44.3)
IPD/OPD (%)	10 (6.5)/ 145 (93.6)	10 (2.6)/ 372 (97.4)	**0.034**	20 (3.7)/ 517 (96.3)
Clinical diagnosis				
Suspected dengue	5 (3.2)	7 (1.8)	0.150	12 (2.2)
Undifferentiated fever	146 (94.2)	372 (97.4)		518 (96.5)
Others	4 (2.6)	3 (0.8)		7 (1.3)
Presence of signs and symptoms (%)				
Retro-orbital pain	72 (46.5)	152 (39.8)	0.156	224 (41.7)
Rash	27 (17.4)	43 (11.3)	0.055	70 (13.0)
Muscle pain	90 (58.1)	213 (55.8)	0.625	303 (56.4)
Joint pain	93 (60.0)	215 (56.3)	0.430	308 (57.4)
Fatigue/weakness	136 (87.7)	342 (89.5)	0.549	478 (89.0)
Headache	125 (80.7)	279 (73.0)	0.064	404 (75.2)
Neck pain	38 (24.5)	86 (22.5)	0.618	124 (23.1)
Ear pain	25 (16.1)	57 (14.9)	0.724	82 (15.3)
Nasal congestion	88 (56.8)	220 (57.6)	0.862	308 (57.4)
Rhinorrhea	88 (56.8)	214 (56.0)	0.873	302 (56.2)
Sore Throat	65 (41.9)	176 (46.1)	0.382	241 (44.9)
Cough	92 (59.4)	252 (66.0)	0.148	344 (64.1)
Sputum production	47 (30.3)	131 (34.3)	0.376	178 (33.2)
Difficulty of breathing	20 (12.9)	57 (14.9)	0.545	77 (14.3)
Nausea & vomiting	71 (45.8)	200 (52.4)	0.169	271 (50.5)
Diarrhea	42 (27.1)	97 (25.4)	0.683	139 (25.9)
Abdominal pain	68 (43.9)	162 (42.4)	0.756	230 (42.8)
Flushed face	52 (33.6)	103 (27.0)	0.127	155 (28.9)
Gum bleeding	5 (3.2)	8 (2.1)	0.440	13 (2.4)
Nose bleeding	14 (9.0)	46 (12.0)	0.316	60 (11.2)
Flushed face	52 (33.6)	103 (27.0)	0.127	155 (28.9)

Values are N (col. %) unless otherwise noted

### Dengue positive cases and clinical characteristics

More than 50% (*n* = 285) of our subjects were < 14 years-of-age. Most patients sought SCH for care, on average, 3.5 days since onset of fever (Table 1). Less than 5% of patients self-reported to have had previous dengue infections, however, this was not validated with medical records. Non-dengue cases (48%) were more likely to receive yellow fever vaccine, compared to dengue-positive cases (36%). Hospitalization was not common, but there were more hospitalized patients in dengue-positive cases (7%), compared to non-dengue patients (3%). Based on univariable associations, dengue-positive patients showed 2.6 times (95% C.I: 1.1–6.3) increased odds to be hospitalized and 1.7 (95% C.I: 1.1–2.5) times increased odds of not having received YF vaccination, compared to non-dengue patients (Table 2).

**Table 2:** Univariable analyses showing significant indicators and unadjusted odds ratios of dengue positive patients compared to non-dengue patients identified in the passive surveillance in Medellin

Characteristics	Dengue positive vs. Non-dengue patients		
	OR	95% CI	*p*-Value
Female (*ref.* male)	0.95	0.65–1.39	0.795
Age (years)			0.867
1–4	Ref	–	
5–9	0.94	0.52–1.71	
10–24	1.79	0.65–1.90	
25–45	2.00	0.72–2.34	
45–65	1.62	0.55–1.92	
Treated at IPD (*ref.* OPD)	**2.57**	**1.05–6.29**	**0.040**
No/unknown yellow fever vaccination (*ref.* vaccinated)	**1.67**	**1.14–2.46**	**0.009**
Fever duration prior to visit			**0.002**
1–2 days	Ref	–	
3–4 days	0.69	0.44–1.09	
5–7 days	1.60	0.99–2.58	
Clinical diagnosis			0.176
Undifferentiated fever	Ref	–	
Suspected dengue	1.82	0.57–5.83	
Non-dengue	3.40	0.75–15.37	
Temperature ≥ 38.0°c at presentation (ref. < 38.0°c)	0.79	0.52–1.22	0.287

Over 90% of patients were clinically diagnosed with undifferentiated fever, prior to lab-confirmation, in both dengue-positive and non-dengue cases, with dengue rarely suspected (Table 1). Furthermore, from 525 patients with no dengue suspicion (clinical diagnosis other than dengue), 150 (28.6%) were later found to be dengue-positive. In terms of symptomatic presentation, there was no significant difference between dengue-positive and non-dengue cases.

Among those acute serum samples tested with RT-PCR (*n* = 173), 8.67% (15/173) were PCR-positive, with serotypes DENV-3 (7/15), DENV-4 (7/15), and DENV-1(1/15), detected (Table 3). Viral isolation assays on C6/36 cells were conducted with these 15 positive samples, in addition to 20 samples which tested negative by RT-PCR but positive with the NS1 test. There was a total of 17 samples found positive for both tests, with 4 samples found negative for isolation and positive by RT-PCR in the serum and 5 samples found positive by the isolation (1 DENV-1; 3 DENV-3; and 1 DENV-4) but with negative results in the RT-PCR from the serum. The most prevalent serotype isolated was DENV-3 (9/17), followed by DENV-4 (7/17) and DENV-1 (1/17), however, complete envelope gene sequence was obtained from 8 out of 9 DENV-3 isolates, 5 out of 7 DENV-4 isolates and from the DENV-1 isolate.

**Table 3:** Laboratory diagnosis among patients with RT-PCR confirmation and virus isolation in the hospital-based fever surveillance established in Medellin, Colombia in 2012–2014

Characteristics		Samples processed by PCR	Samples by virus isolation	No. samples by PCR and/or virus isolation	
				2012	2013
RT-PCR	No. processed	173	35	23	135
	Confirmed				
	Negative	158 (91.33)	18	21	123
	Positive	15 (8.67)	17*	2	14
	Serotype 1	1 (6.67)	1 (5.88)	0	1
	Serotype 3	6 (40.0)	9 (52.94)	2	6
	Serotype 4	8 (53.33)	7 (41.18)	0	7

*one positive case of DENV 3 found in January 2014

By the calendar year, DENV-3 was prevalent in 2012, while the majority were DENV-3 and -4 in 2013 (Table 3). Overall, the number of samples with complete serotyping was small.

### Sequencing and phylogenetic analysis

The phylogenetic reconstructions showed that the DENV-1 isolate (UW22) belongs to the genotype V or American/African genotype (Fig. S1), all 8 DENV-3 isolates belong to the genotype III (Fig. S2), and all 5 DENV-4 isolates to the genotype II sub-lineage IIb (Fig. S3). Downsizing of each serotype dataset and subsequent root-to-tip analysis on TempEST showed enough temporal signal supporting the use of a molecular clock analysis to establish phylogenetic relationship within genotypes (Fig. S4). For the model selection process required for the Bayesian phylogenetic reconstructions, the generalized time reversible plus invariant and discrete gamma model (GTR + I + G) was the best fit nucleotide substitution model for DENV-1 and DENV-3 datasets while Tamura and Nei plus invariant and discrete gamma model (TrN + I + G) was for DENV-4 (Table S1). Based on the comparison of all eight combinations of models (two molecular clock models and four demographic growth models), the strict molecular clock and Bayesian skyline growth was the best fit model combination for DENV-1, the uncorrelated lognormal (UCLN) relaxed clock and constant size growth for DENV-3, and the UCLN relaxed clock and Bayesian skyline growth for DENV-4 (Table S2). Estimation of mean nucleotide substitution rates were similar to those reported for the same genotypes circulating in the Americas [26–28]: 7.31 × 10^− 4^ substitution/site/year (95% highest posterior density interval – HPD: 6.467 × 10^− 4^–8.252 × 10^− 4^), 1.02 × 10^− 3^ substitutions/site/year (95% HPD: 8.41 × 10^− 4^–1.18 × 10^− 3^), and 1.06 × 10^− 3^ substitutions/site/year (95% HPD: 8.66 × 10^− 4^–1.25 × 10^− 3^) for DENV-1, DENV-3, and DENV-4 respectively.

**Fig. 5:**
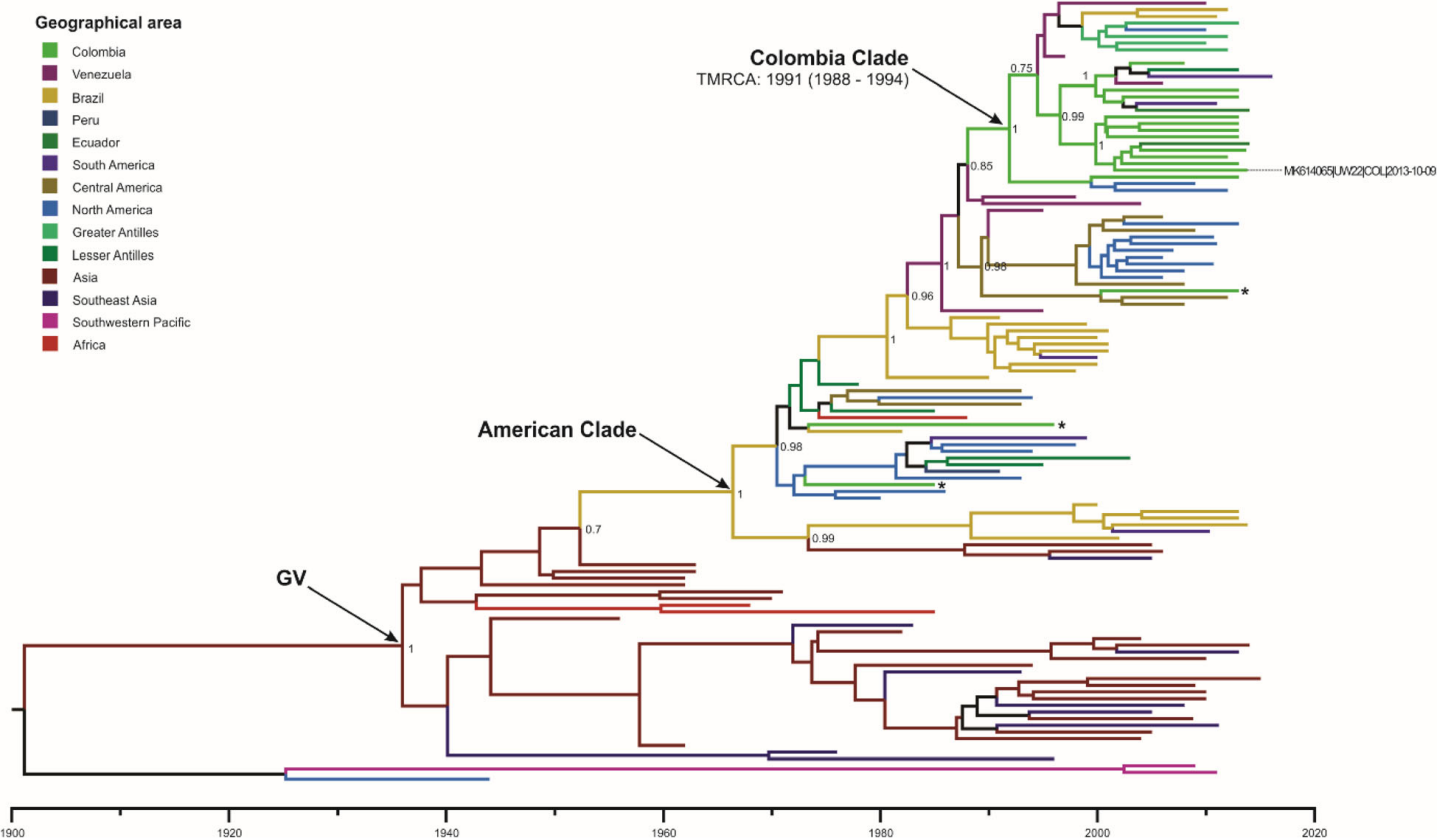
Maximum clade credibility tree of DENV-1 – genotype V

DENV-1 isolate in this study (UW22) clustered with most of the included Colombian sequences in a single monophyletic group denoted here as “Colombia Clade” which had an estimated TMRCA around 1991 (95% HPD: 1988–1994) (Fig. 5). This clade included strains from the departments of Santander (*n* = 10) and Sucre (*n* = 1) in Colombia isolated between 2008 and 2013, but also sequences from Venezuela (1997–2010), Brazil (2011–2012), Ecuador (2014), the Greater Antilles (Puerto Rico/ Jamaica/Haiti, 2010–2013), the Lesser Antilles (Barbados, 2013), South America (Argentina, 2010–2016), and North America (Mexico/United States of America, 2009–2012). Despite the fact that the phylogenetic reconstruction suggests multiple introductions of DENV-1 to Colombia (there are Colombian strains isolated in 1985 and 1996 that were more related to strains from Brazil, the Lesser Antilles, and North America), the “Colombia Clade” might represent the current circulating lineage of this serotype in Colombia because it includes only strains with more recent isolation. Ancestral nodes to the “Colombia Clade” contain sequences from Venezuelan strains isolated in 1998 and 2004, suggesting this country as the origin of the currently circulating lineage in Colombia.

The tree was constructed with 114 strains from the genotype V and 3 strains from the genotype I as outgroup. Tips are color-coded according to the geographic area of origin and labelled tip corresponds to the strain isolated in the present study. Posterior probabilities are indicated on the nodes of relevant clades. Mean estimated time to the most recent common ancestor (TMRCA) with corresponding 95% highest posterior density interval (HPD) is indicated for relevant clade. *: Colombian strains that were isolated in 1985 (AF425616), 1996 (AF425617), and 2013 (KY818080).

**Fig. 6:**
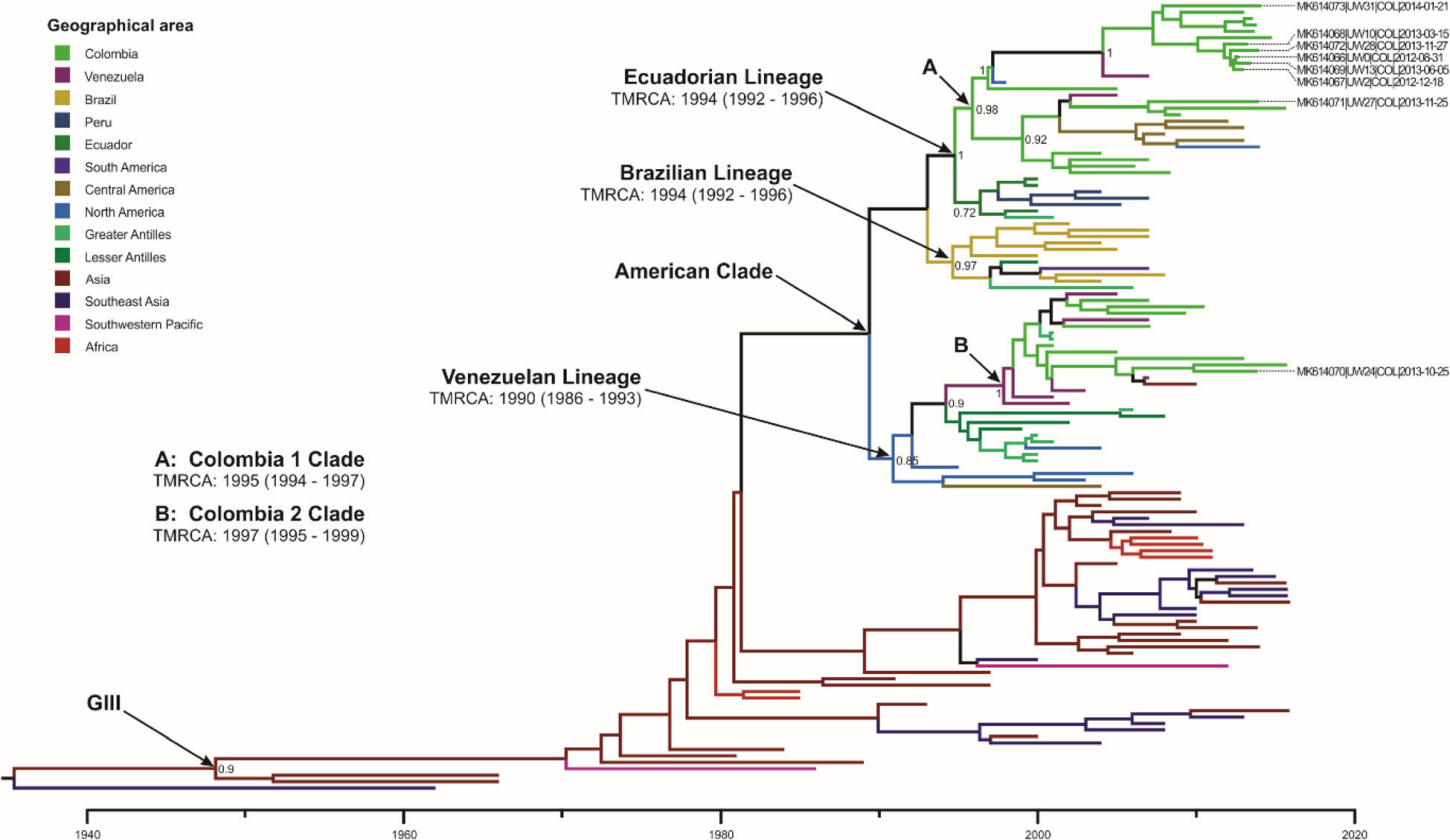
Maximum clade credibility tree of DENV-3 – genotype III

Strains of DENV-3 isolates were found clustering in two different monophyletic clades formed mainly by Colombian strains (Fig. 6). These two clades were denoted here as “Clade A” and “Clade B”, and were identified to be within the previously reported Ecuadorian or Peru-Ecuador and Venezuelan [29, 30] lineages, respectively. “Clade A” included seven strains isolated in this study (2012–2013) along with strains from the departments of Santander (*n* = 5, 2004–2014), Antioquia (*n* = 2, 2007–2009) Sucre (*n* = 3, 2013), Norte de Santander (*n* = 1, 2005), Boyaca (*n* = 1, 2015), and Bolivar (*n* = 1, 2006) in Colombia. This clade also included strains from Venezuela (2005–2007), Central America (Honduras/Nicaragua, 2008–2013), and North America (United States of America, 1998–2014), with an estimated TMRCA around 1995 (95% HPD: 1994–1997). Ancestral nodes to this clade included strains from Ecuador (2000), Peru (2004–2007), and the Greater Antilles (Cuba, 2001) which suggest two possible introduction origins: from the Greater Antilles or the south of the country.

The tree was constructed with 122 strains from the genotype III and 1 strain from the genotype II as outgroup. Tips are color-coded according to the geographic area of origin and labelled tips correspond to the strains isolated in the present study. Posterior probabilities are indicated on the nodes of relevant clades. Mean estimated time to the most recent common ancestor (TMRCA) with corresponding 95% highest posterior density interval (HPD) is indicated for relevant clade.

The UW24 strain isolated in 2013 clustered within the “Clade B” along with strains from Santander (*n* = 5, 2001–2013), Antioquia (*n* = 2, 2007–2015), and Norte de Santander (*n* = 1, 2005) in Colombia. Within this clade, there were strains from Venezuela (2001–2010), and the Greater Antilles (Cuba, 2001) with an estimated TMRCA around 1997 (95% HPD: 1995–1999). Ancestral nodes included strains from the Greater Antilles (Puerto Rico, 2000–2006), the Lesser Antilles (Saint Kitts and Nevis/Aruba/Grenada, 1999–2008), North America (Mexico/United States of America, 1995–2006), and Central America (Belize, 2004), which suggest introduction from the Caribbean or Central America. The estimated TMRCA of these two different clades and that most of the strains included were of relatively recent isolation (2001–2015) support the evidence of a simultaneous introduction of these two lineages during the late 1990s and their continued circulation in Colombia thereafter.

**Fig. 7:**
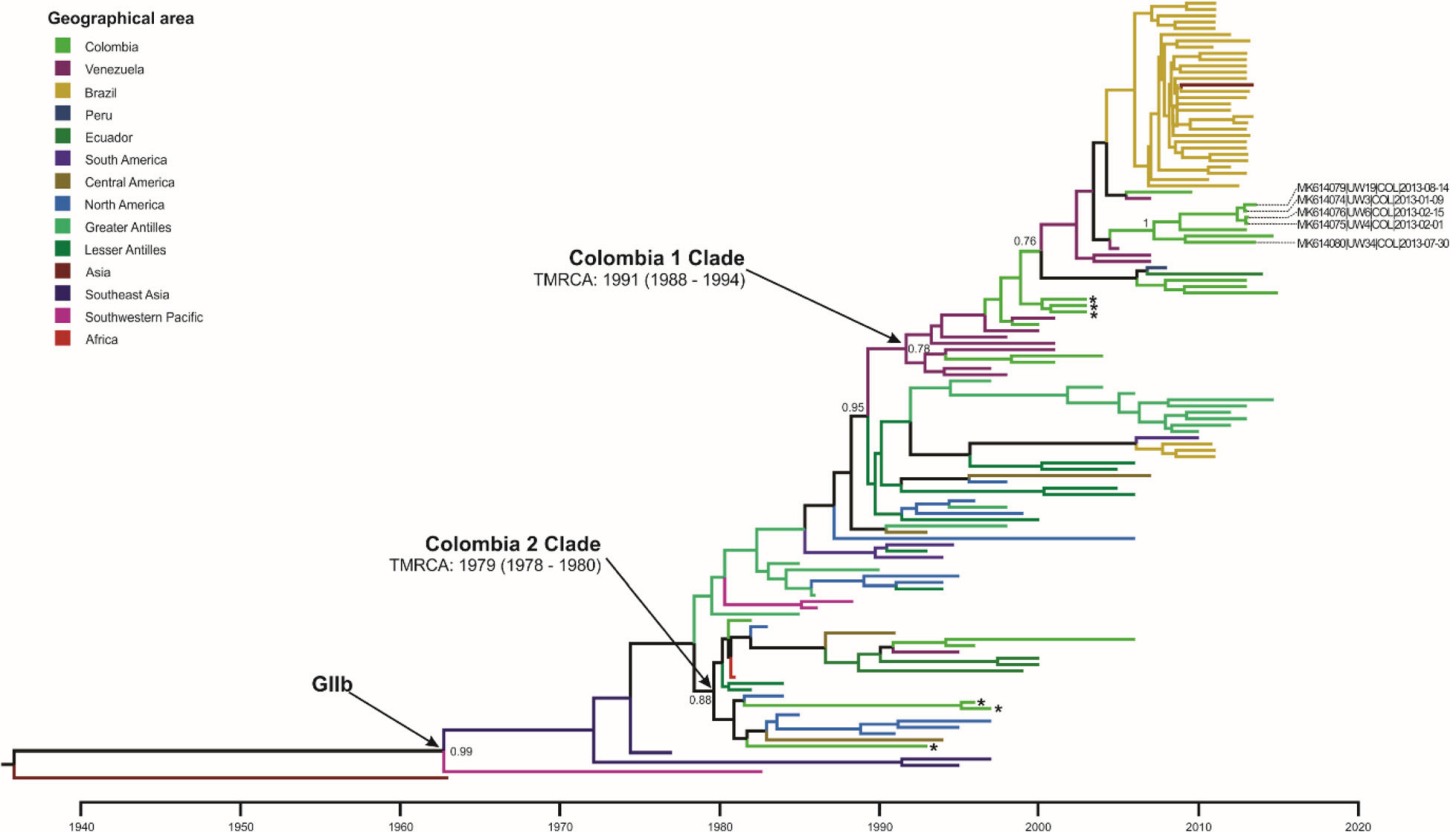
Maximum clade credibility tree of DENV-4 – genotype IIb

Our phylogenetic reconstructions showed two monophyletic clades containing Colombian DENV-4 strains denoted here as “Colombia 1 Clade” and “Colombia 2 Clade” (Fig. 7). All five strains of DENV-4 in this study (2013) were grouped within the “Colombia 1 Clade” along with strains from Santander (*n* = 8, 2000–2014), Antioquia (*n* = 3, 2003), and Cesar (*n* = 1, 2015) in Colombia. This clade also included strains from Venezuela (1997–2007), Brazil (2010–2013), Peru (2008), and Ecuador (2014). The estimated TMRCA was around 1991 (95% HPD: 1988–1994) with ancestral nodes containing strains from Venezuela (1997–1998) suggesting an introduction from this country. The “Colombia 2 Clade” contains Colombian strains isolated before 1997 from the department of Antioquia and others with no precise information on location, however one strain (JF804052) with no available information of location and isolated in 2006 is also included in this group. The clade also contains strains from Venezuela (1995), Ecuador (1999–2000), Central America (El Salvador/Honduras, 1991–1994), the Lesser Antilles (Trinidad and Tobago, 1982–1984), and North America (Mexico, 1984–1997), with an estimated TMRCA around 1979 (95% HPD: 1978–1980).

The tree was constructed with 123 strains from the genotype IIb and 1 strain from the genotype I as outgroup. Tips are color-coded according to the geographic area of origin and labelled tips correspond to the strains isolated in the present study. Posterior probabilities are indicated on the nodes of relevant clades. Mean estimated time to the most recent common ancestor (TMRCA) with corresponding 95% highest posterior density interval (HPD) is indicated for the relevant clade. *: Colombian strains from the department of Antioquia that were isolated in the 1990s and during 2003.

## Discussion

During the period of our surveillance between November 2011 and February 2014, there was no reported epidemic in Medellin. Nonetheless, more than 1 in 4 febrile patients with non-localizing signs were dengue-positive. This rate was similar to what was previously reported. In an observational study conducted in Cali among 235 febrile elderly patients in 2011–2014, 18% (*n* = 43) of febrile patients were diagnosed with dengue [31]. Despite the difference in age groups, the estimates are similar to our finding.

Despite the high proportion of dengue-positive cases in this study, clinical diagnosis of suspected dengue was rare. Of dengue-positive patients (*n* = 155), only 5 patients were initially diagnosed with suspected dengue, with the rest 146 clinically diagnosed with undifferentiated fever. After the 2nd visit, of dengue-positive patients (*n* = 155), 40 (26%) patients had dengue as the final diagnosis, but still undifferentiated fever was the most common final diagnosis (*n* = 112). Even during the times of absence of known dengue epidemics, given the hyperendemicity of dengue in Colombia, clinicians may need to consider dengue more frequently as a clinical diagnosis [32].

In the Americas, including Colombia, dengue is known to affect individuals across all ages [1, 9]. In our data, with dengue-positive cases found in all age groups, the mean age of dengue-positive cases was 20.6 years. Furthermore, we observed a higher proportion of dengue cases in children and young adults < 35 years-of-age, also consistent with previous reports [1].

In terms of hospitalization, only 7% of dengue-positive patients were hospitalized in our surveillance. This is similar to other studies, such as assessment of impact of a dengue case management system on hospitalization in 2003–2004 in primary care setting in Santander, Colombia, where 44 hospitalizations were reported in 18-week period among 964 dengue-suspected patients [33].

While our data were limited to collect information on severity of illness, there were possible indicators of severity, such as fever duration, IPD (vs. OPD) and secondary (vs. primary) dengue. There was only a small number of hospitalized episodes (20 out of 537 enrolled) and patients reporting hemorrhagic or other warning signs were rare. In addition to absence of common signs of severe dengue found among 20 hospitalized patients, the length of hospitalization of dengue-positive patients was relatively short with the mean duration of 4.38 days, ranging between 3 and 6 days.

Furthermore, based on IgM and/or IgG results, 53 and 101 patients were considered to have had secondary and primary dengue infection, respectively. While Colombia is considered highly dengue-endemic, there were more primary dengue cases than secondary dengue cases in the studied population. Of 10 dengue-positive cases among 20 hospitalized patients, 8 of them would be classified as secondary dengue cases. While the majority of hospitalized dengue patients showed to have secondary dengue than primary dengue infections, interpretation may be limited due to a small sample size. Our laboratory data were incomplete in terms of paired IgM and/or IgG results (i.e., only acute results available) for classification of secondary vs. primary dengue cases. Nonetheless, our data suggest mildness of dengue disease in Medellin at these times without large epidemics.

Taking place prior to the Chikungunya epidemic in 2014 and Zika epidemic in 2015, the molecular epidemiology of DENV was captured in the study without interference of other arboviruses and could be used as a basis to observe variation [34, 35]. Also, genetic diversity of DENV isolates and relatedness to other isolates were documented. DENV-1 isolate belonging to the genotype V, is a well-established variant in the Americas and previously reported to have circulated in Colombia since 1977 [36]. This is also consistent with the DENV serotypes and genotypes sequenced in Sanofi Pasteur’s phase 3 efficacy (CYD15) trial in Colombia [37]. It was closely related to strains from other parts of Colombia, within a single clade intermixed with strains from countries, like Venezuela, Ecuador, and Brazil. There have been reports of the possible current circulation of two DENV-1 – Genotype V putative lineages in Colombia [36] one of them exclusively formed by Colombian isolates, based on phylogenetic reconstructions using partial segments of E gene. Despite the caveat of having only one isolate sequenced for DENV-1, there is no evidence in this study supporting the presence of these two putative lineages (at least not with the sequences included in the study) and this finding is consistent with recent reports of evolutionary history of DENV-1 in Colombia [28].

DENV-3 isolates obtained belonged to the genotype III, consistent with previous reports [29, 38] and findings from sequencing data in the dengue vaccine phase 3 trial in Colombia [37]. Some studies suggested that most recent DENV-3 viruses circulating in South America have accumulated enough genetic variation that caused formation of clusters totally different from those described in Central America [39]. Also, there are suggestions of possible circulation of an Asian-origin genotype I [38] in La Guajira (North), Guaviare (Southeast), and Huila (Southwest) in Colombia, but there was no evidence of circulation of this genotype in the study. Two different lineages of genotype III viruses were found to overlap in space and time during the study which could be an effect of two independent transmission chains since their official re-introduction in Colombia in 2001 [40] due to complete susceptibility of the population to this serotype after 24 years with no circulation. Our TMRCA estimations suggest that introduction of both lineages to Colombia occurred during the late 1990’s (1995 for Clade A and 1997 for Clade B), which was earlier than the first official report of circulation, indicating ongoing local circulation of DENV-3 before its detection and possibly causing an increase in the number of infections during that period; similar TMRCA estimations have been reported previously, supporting the finding of an earlier introduction into the country [41]. This simultaneous circulation has been reported previously in Medellin [42], and more recently in Santander and Valle del Cauca, in Colombia [28]. Additionally, it has been documented that the most probable ancestral locations for the clades we denoted here as “Clade A” and “Clade B” were the Greater Antilles and Central America, respectively [28].

All DENV-4 isolates belonged to genotype II, to the IIb linage, which is a variant mainly circulating in the Caribbean. It is consistent with the DENV serotypes and genotypes sequenced in Sanofi Pasteur’s phase 3 efficacy trial in Colombia [37]. Our isolates showed to group with those that were more recently isolated in this region, forming a cluster different from other Colombian strains isolated before 1997. Recently, circulation of this genotype has been reported in at least three other departments of Colombia (Santander, Valle del Cauca, and Cesar) and is reported to have caused at least two introductions in the country [28]. Phylogeographic analysis revealed that the genotype II was circulating along with genotype I in Brazil during 2010–2011 [43]. However, our study does not provide evidence of the circulation of this additional genotype. Nunes and coworkers described multiple introductions of genotype II to Brazil in the last decade, at least three of them from Colombia and Venezuela, which corresponds with the finding of multiple clusters within the genotypes [43].

Commonly, the phylogenetic reconstructions of DENV have been performed with the complete E gene, as in this study, creating an imbalance in the amount of available sequences from more ancestral origins for analysis. There are reports supporting that phylogenetic information obtained from the complete E gene is representative and similar to what is generally obtained with complete genome sequences of the virus [41, 44–46].

Mainly due to the variations on viremia levels, DENV genetic sequencing generally requires isolation of the virus on cell monolayers to have enough genetic material. In our study, isolated viruses were used for sequencing and there may be the possibility of mutations induced by the cell passaging, however the amount of passages was maintained low to reduce this effect.

The study has limitations. Due to resource constraints, this study continued for 28 months and was conducted in one area of Medellin. Also, although there was a large number of samples collected in the study, only a limited number of samples were tested with RT-PCR and virus isolation. Samples to be tested with RT-PCR and virus isolation were selected based on paired ELISA and/or RDT positive results, and there may be selection bias associated, resulting in limited interpretation of our findings from the phylogenetic analysis.

Furthermore, the rate of detection of dengue positivity was lower by PCR than serological methods based on IgM/IgG ELISA in our study. Degradation of RNA could have contributed to the lower detection rate of PCR. However, PCR was performed in two locations: one in Medellin soon after sample collection and another in University of Wisconsin, and results were comparable. One noteworthy point is the duration of fever prior to hospital visit (enrollment). Given that PCR works better in the early febrile phase [47], the fact that 70% of our patients were enrolled in the study 3 days or later since onset of fever could have contributed to the lower rate of case detection by RT-PCR.

Another important potential source of bias in our study is under-ascertainment of the community residents with relevant symptoms. Despite that the SCH is the main hospital of the comuna, our study may have missed those eligible patients seeking care at other healthcare providers than our facility. Thus, we may have missed other mild fever episodes. Therefore, we recognize limited generalizability of our data to represent the general population of the country due to these limitations.

In the surveillance, among these 155 dengue-positive patients, 99 cases were dengue-probable cases. Given the DENV endemicity in the country, dengue-probable cases, along with confirmed cases, are reported as part of dengue surveillance in the national system in Colombia. Furthermore, this surveillance was conducted in the same catchment area population as the repeated community-based serological surveys measuring seroprevalence in the same study population [48]. The seroprevalence study reported 61% of seroprevalance with 8.7% of sero-conversion per 1000 person-months [48]. Without major epidemics during our study period, the estimated proportion of dengue-positive cases among febrile patients as well as the seroprevalence estimate and sero-conversion rate all support high transmission and burden of DENV in the study area.

## Conclusions

Our findings confirm that there is considerable dengue burden in Santa Cruz comuna during non-epidemic years, with the majority with mild illness. Conducted prior to Chikungunya epidemic in 2014 and Zika epidemic in 2015, the study findings support genetic diversity of DENV isolates and their relatedness to isolates from other nearby countries. Now with emergence of new epidemics, our results support the need for continued surveillance and monitoring of dengue and other arboviruses.

## Supplementary information

**Additional file 1: S1.** Checklist: STROBE Checklist. **Figure S1**. DENV-1 Maximum Likelihood tree and root-to-tip regression. A) Linear regression of root-to-tip divergence and dates of isolation indicating the slope and R-squared value for temporal signal evaluation. Each datapoint is color-coded based on the corresponding genotype within DENV-1. B) Maximum likelihood tree with 954 full-length E-gene sequences (1485 nt) representing the five genotypes reported for DENV-1. Tips are colored by corresponding genotype and labelled tip indicate the strain obtain in this study. The tree was rooted with the sequence DENV2-NGC strain as outgroup (GenBank: KM204118) and the sequence names are coded as GenBank accession|ISO-3166 Alpha-3 country code|Date of isolation. **Figure S2**. DENV-3 Maximum Likelihood tree and root-to-tip regression. A) Linear regression of root-to-tip divergence and dates of isolation indicating the slope and R-squared value for temporal signal evaluation. Each datapoint is color-coded based on the corresponding genotype within DENV-3. B) Maximum likelihood tree with 553 full-length E-gene sequences (1479 nt) representing the five genotypes reported for DENV-3. Tips are colored by corresponding genotype and labelled tips indicate the strains obtain in this study. The tree was rooted with the sequence DENV-1-Hawaii strain as outgroup (GenBank: KM204119) and the sequence names are coded as GenBank accession|ISO-3166 Alpha-3 country code|Date of isolation. **Figure S3**. DENV-4 Maximum Likelihood tree and root-to-tip regression. A) Linear regression of root-to-tip divergence and dates of isolation indicating the slope and R-squared value for temporal signal evaluation. Each datapoint is color-coded based on the corresponding genotype within DENV-4. B) Maximum likelihood tree with 867 full-length E-gene sequences (1485 nt) representing the four genotypes reported for DENV-4. Tips are colored by corresponding genotype and labelled tips indicate the strains obtain in this study. The tree was rooted with the sequence DENV2-NGC strain as outgroup (GenBank: KM204118) and the sequence names are coded as GenBank accession|ISO-3166 Alpha-3 country code|Date of isolation. **Figure S4**. Root-to-tip analysis for identified genotypes. Linear regression of root-to-tip divergence and date of isolation for the E-gene of DENV-1 (GV), DENV-3 (GIII) and DENV-4 (GIIb) to evaluate the temporal structure of datasets. Each plot shows the R-squared value and slope of the black dashed regression line which indicate the substitution rate for these viruses. The linear regression supports the use of these data for molecular clock inferences. Each datapoint is color-coded based on the geographic area of origin. **Table S1**. Nucleotide Substitution model selection. Results for the statistical best fit model selection process with jModelTest for each serotype. **Table S2**. Molecular clock and demographic growth model selection. Marginal likelihoods calculated with path-sampling (PS) and stepping-stone sampling (SS) methods for the combinations of four demographic growth models (constant size, exponential, Bayesian Skyline and Bayesian SkyGrid) and two molecular clock models (strict clock and uncorrelated relaxed clock with log-normal distribution). Bayes factors were calculated against the model combination with the lower marginal likelihood estimation which in all three cases was the constant tree prior and strict clock.

## Data Availability

The dataset supporting the conclusions of this article is included within the article and its additional file. All sequences were deposited into the GenBank database under the following accession numbers (which are parts of the sequence names that are shown in the generated phylogenetic trees): MK614065, MK614073, MK614068, MK614072, MK614066, MK614069, MK614067, MK614071, MK614070, MK614079, MK614074, MK614076, MK614075, MK614080.

## Abbreviations

CI: Confidence Interval
°C: Celsius degrees
CRF: Case Report Form
CYD: Sanofi Pasteur’s phase 3 efficacy trial
DENV: Dengue Virus
DF: Dengue Fever
DHF: Dengue Hemorrhagic Fever
DSS: Dengue Shock Syndrome
DVI: Dengue Vaccine Initiative
ELISA: Enzyme-Linked Immunosorbent Assay
GTR: Generalized time reversible
HPD: Highest Posterior Density
ICF: Informed Consent Form
IgM/IgG: Immunoglobulin type M and type G
IPD: Inpatient Department
IRB: Institutional Review Board
IVI: International Vaccine Institute
ML: Maximum Likelihood
MCC: Maximum Clade Credibility trees
MCMC: Markov Chain Monte Carlo
OPD: Outpatient Department
PS: Path-sampling
RDT: Rapid Diagnostic Test
PECET: Programa de Estudio y Control de Enfermedades Tropicales
RT-PCR: Reverse Transcriptase-Polymerase Chain Reaction
SCH: Santa Cruz Hospital
SD: Standard Diagnostics
SS: Stepping-stone Sampling
TMRCA: Time of Most Recent Common Ancestor
TrN: Tamura and Nei
UCLN: Uncorrelated Lognormal
URI: Upper Respiratory Illness
YF: Yellow fever
