# Supplementary material for: Epidemiology and genetic diversity of circulating dengue viruses in Medellin, Colombia: a fever surveillance study
Source: BMC Infect Dis. 2020 Jul 2;20:466. doi: 10.1186/s12879-020-05172-7 (PMC7331258; doi:10.1186/s12879-020-05172-7)
Supplement: Supplementary file 1 — Additional file 1: S1. Checklist: STROBE Checklist. Figure S1. DENV-1 Maximum Likelihood tree and root-to-tip regression. A) Linear regression of root-to-tip divergence and dates of isolation indicating the slope and R-squared value for temporal signal evaluation. Each datapoint is color-coded based on the corresponding genotype within DENV-1. B) Maximum likelihood tree with 954 full-length E-gene sequences (1485 nt) representing the five genotypes reported for DENV-1. Tips are colored by corresponding genotype and labelled tip indicate the strain obtain in this study. The tree was rooted with the sequence DENV2-NGC strain as outgroup (GenBank: KM204118) and the sequence names are coded as GenBank accession|ISO-3166 Alpha-3 country code|Date of isolation. Figure S2. DENV-3 Maximum Likelihood tree and root-to-tip regression. A) Linear regression of root-to-tip divergence and dates of isolation indicating the slope and R-squared value for temporal signal evaluation. Each datapoint is color-coded based on the corresponding genotype within DENV-3. B) Maximum likelihood tree with 553 full-length E-gene sequences (1479 nt) representing the five genotypes reported for DENV-3. Tips are colored by corresponding genotype and labelled tips indicate the strains obtain in this study. The tree was rooted with the sequence DENV-1-Hawaii strain as outgroup (GenBank: KM204119) and the sequence names are coded as GenBank accession|ISO-3166 Alpha-3 country code|Date of isolation. Figure S3. DENV-4 Maximum Likelihood tree and root-to-tip regression. A) Linear regression of root-to-tip divergence and dates of isolation indicating the slope and R-squared value for temporal signal evaluation. Each datapoint is color-coded based on the corresponding genotype within DENV-4. B) Maximum likelihood tree with 867 full-length E-gene sequences (1485 nt) representing the four genotypes reported for DENV-4. Tips are colored by corresponding genotype and labelled tips indicate the strains obtain in [file 12879_2020_5172_MOESM1_ESM.docx]

**Supporting Information**

*Figures*

Title: Epidemiology and genetic diversity of circulating dengue viruses in Medellin, Colombia: A fever surveillance study

Jacqueline K. Lim^a^, Mabel Carabali^a b^, Erwin Camacho^c^, Diana Carolina Velez^d^, Andrea Trujillo^d^, Jorge Egurrola^d^, Kang-Sung Lee^a^, Ivan Dario Velez^d^, Jorge E Osorio^e^

^a^Dengue Vaccine Initiative, International Vaccine Institute, SNU Research Park, Gwankak-ro 1, Gwanak-gu, Seoul, Republic of Korea, 08826; ^b^McGill University, 845 Sherbrooke St., W, Montreal, Quebec H3A 0G4, Canada; ^c^Investigaciones Biomedicas, Universidad de Sucre, Cra 28 # 5-267, Barrio Puerta Roja - Sincelejo (Sucre), Colombia; ^d^Programa de Estudio y Control de Enfermedades Tropicales (PECET), Universidad de Antioquia, calle 67 No. 53 – 108, Medellín, Antioquia, Colombia; and ^e^Department of Pathobiological Sciences, University of Wisconsin, 500 Lincoln Dr., Madison, WI, 53706

Corresponding author: Jacqueline Kyungah Lim

Corresponding address: Dengue Vaccine Initiative, International Vaccine Institute, SNU research park, Gwanak-ro 1, Gwanak-gu, Seoul, Republic of Korea, 08826

Email: kalim@ivi.int

**Fig. S1. DENV1 Maximum Likelihood tree and root-to-tip regression.**

**A)** Linear regression of root-to-tip divergence and dates of isolation indicating the slope and R-squared value for temporal signal evaluation. Each datapoint is color-coded based on the corresponding genotype within DENV1. **B)** Maximum likelihood tree with 954 full-length E-gene sequences (1485 nt) representing the five genotypes reported for DENV1. Tips are colored by corresponding genotype and labelled tip indicate the strain obtain in this study. The tree was rooted with the sequence DENV2-NGC strain as outgroup (GenBank: KM204118) and the sequence names are coded as GenBank accession|ISO-3166 Alpha-3 country code|Date of isolation.


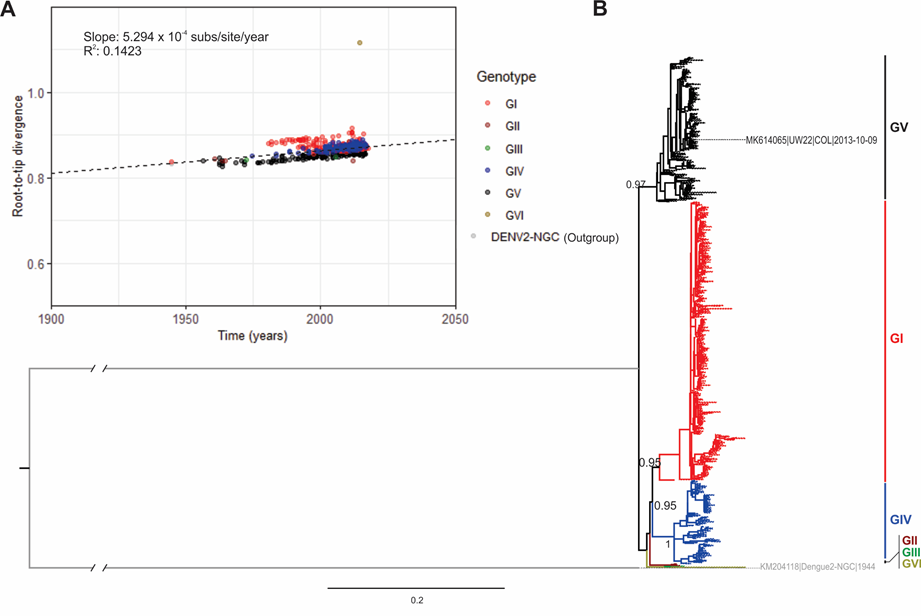


**Fig. S2. DENV3 Maximum Likelihood tree and root-to-tip regression.**

**A)** Linear regression of root-to-tip divergence and dates of isolation indicating the slope and R-squared value for temporal signal evaluation. Each datapoint is color-coded based on the corresponding genotype within DENV3. **B)** Maximum likelihood tree with 553 full-length E-gene sequences (1479 nt) representing the five genotypes reported for DENV3. Tips are colored by corresponding genotype and labelled tips indicate the strains obtain in this study. The tree was rooted with the sequence DENV1-Hawaii strain as outgroup (GenBank: KM204119) and the sequence names are coded as GenBank accession|ISO-3166 Alpha-3 country code|Date of isolation.


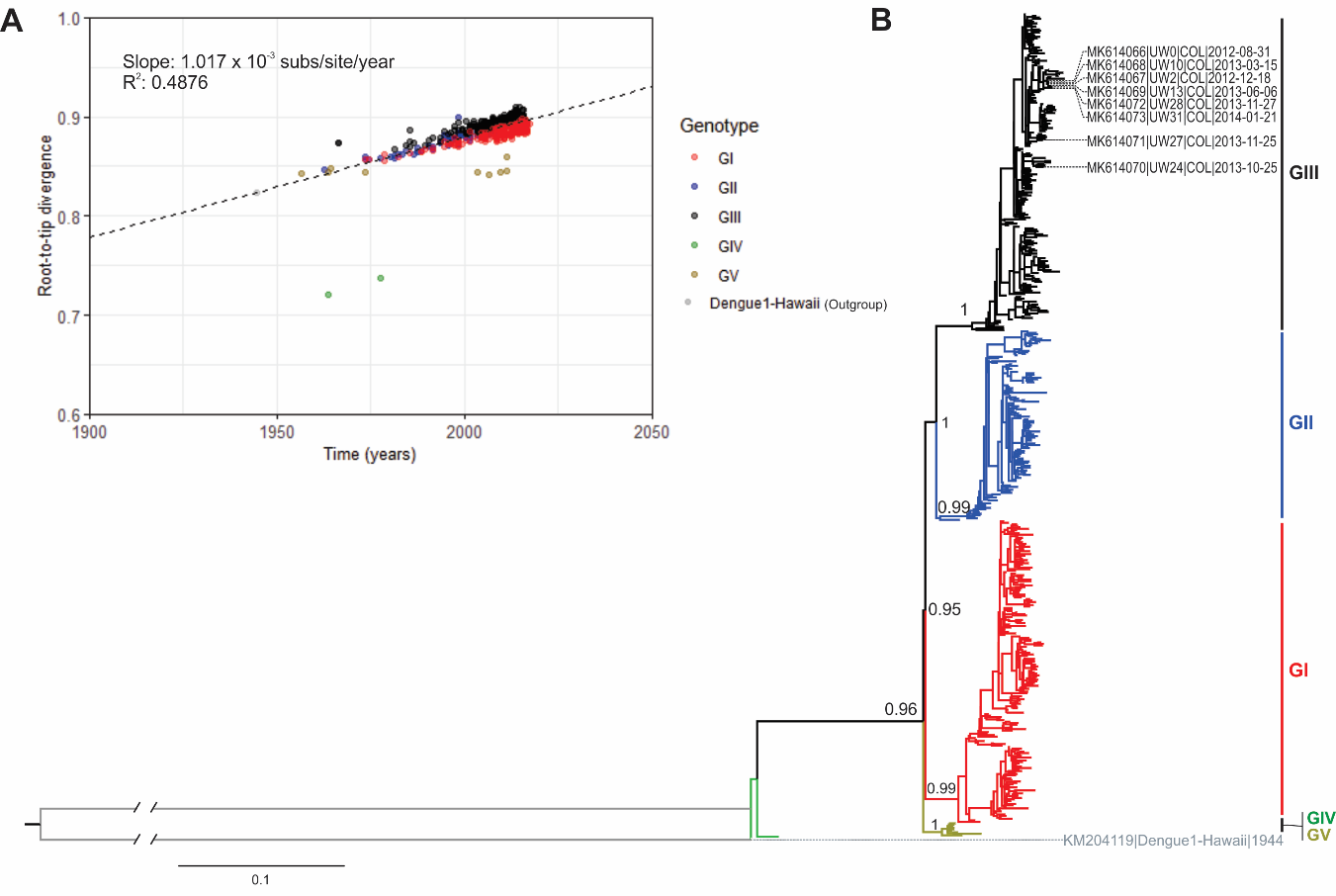


**Fig. S3. DENV4 Maximum Likelihood tree and root-to-tip regression.**

**A)** Linear regression of root-to-tip divergence and dates of isolation indicating the slope and R-squared value for temporal signal evaluation. Each datapoint is color-coded based on the corresponding genotype within DENV4. **B)** Maximum likelihood tree with 867 full-length E-gene sequences (1485 nt) representing the four genotypes reported for DENV4. Tips are colored by corresponding genotype and labelled tips indicate the strains obtain in this study. The tree was rooted with the sequence DENV2-NGC strain as outgroup (GenBank: KM204118) and the sequence names are coded as GenBank accession|ISO-3166 Alpha-3 country code|Date of isolation.


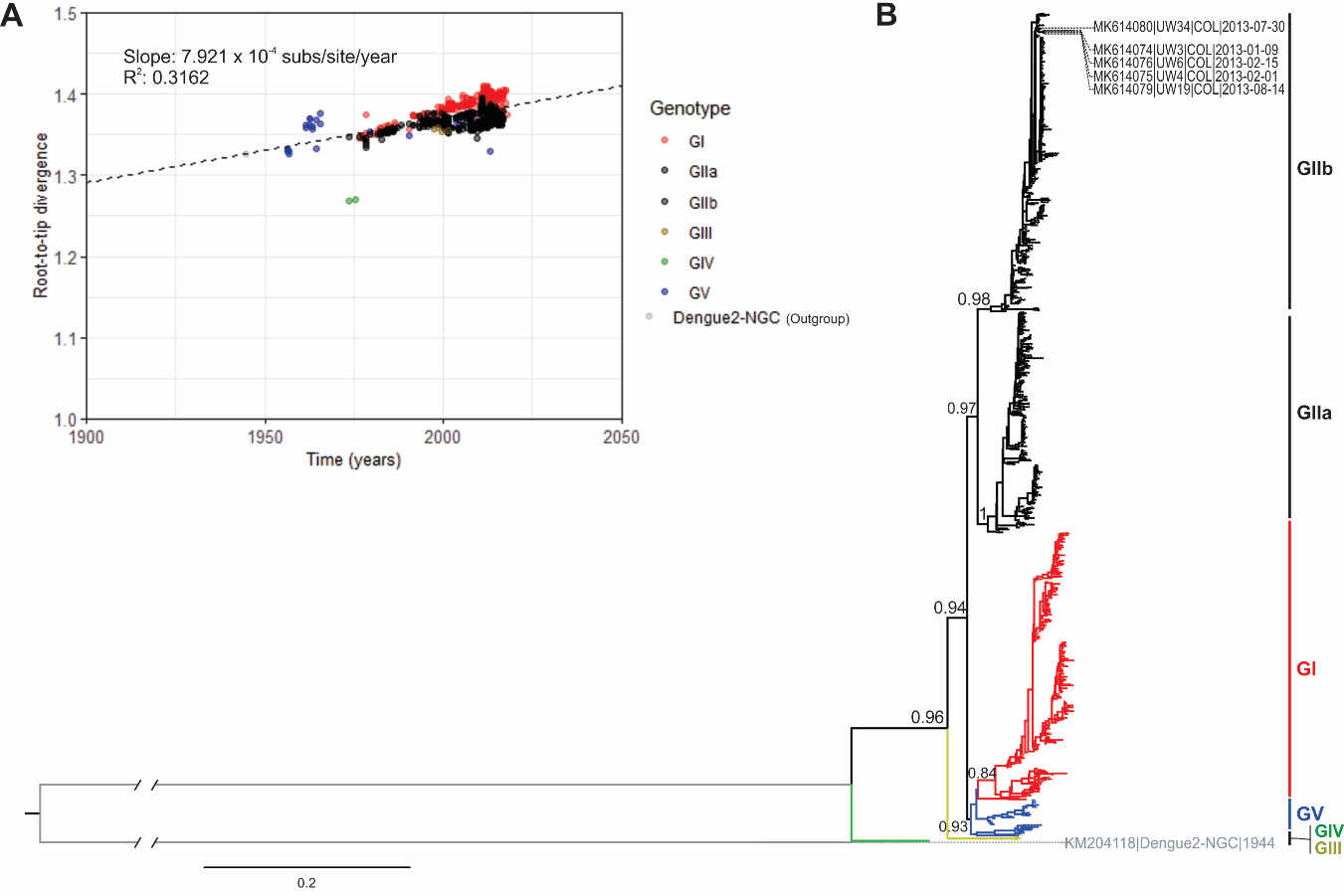


**Fig. S4. Root-to-tip analysis for identified genotypes.**

Linear regression of root-to-tip divergence and date of isolation for the E-gene of DENV1 (GV), DENV3 (GIII) and DENV4 (GIIb) to evaluate the temporal structure of datasets. Each plot shows the R-squared value and slope of the black dashed regression line which indicate the substitution rate for these viruses. The linear regression supports the use of these data for molecular clock inferences. Each datapoint is color-coded based on the geographic area of origin.


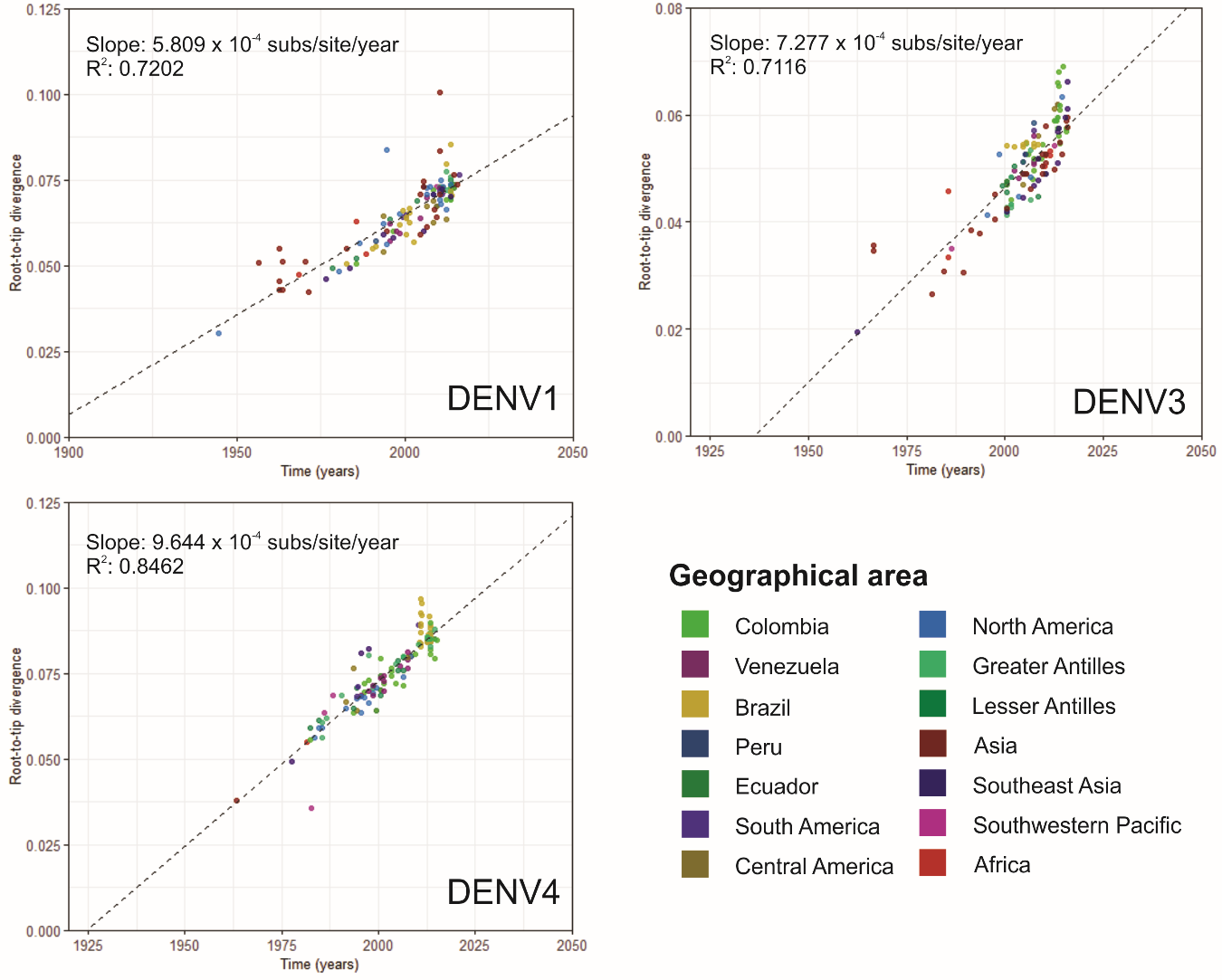


*Tables*

**Table S1. Nucleotide Substitution model selection**

Results for the statistical best fit model selection process with jModelTest for each serotype.

| Serotype | Model | -lnL | K | AIC |
| --- | --- | --- | --- | --- |
| DENV1 | GTR + I + G | 11911.23388 | 234 | 24290.467760 |
| DENV3 | GTR + I + G | 9142.70395 | 230 | 18745.407900 |
| DENV4 | TrN + I + G | 8629.86479 | 251 | 17761.729580 |

**Table S2. Molecular clock and demographic growth model selection**

Marginal likelihoods calculated with path-sampling (PS) and stepping-stone sampling (SS) methods for the combinations of four demographic growth models (constant size, exponential, Bayesian Skyline and Bayesian SkyGrid) and two molecular clock models (strict clock and uncorrelated relaxed clock with log-normal distribution). Bayes factors were calculated against the model combination with the lower marginal likelihood estimation which in all three cases was the constant tree prior and strict clock.

DENV1

|  |  | Skyline | **Skyline** | Exponential | Exponential | Skygrid | Skygrid | Constant | Constant |
| --- | --- | --- | --- | --- | --- | --- | --- | --- | --- |
|  |  | Relaxed | **Strict** | Relaxed | Strict | Relaxed | Strict | Relaxed | Strict |
| PS | Log(marginal likelihood) | -13080 | **-12996** | -13088 | -13136 | -13118 | -13177 | -13117 | -13178 |
|  | Log(Bayes factor) | 98 | **182** | 90 | 42 | 60 | 0 | 61 | - |
| SS | Log(marginal likelihood) | -13080 | **-12835** | -13091 | -13136 | -13119 | -13178 | -13118 | -13178 |
|  | Log(Bayes factor) | 98 | **343** | 88 | 42 | 59 | 0 | 61 | - |

DENV3

|  |  | Skyline | Skyline | Exponential | Exponential | Skygrid | Skygrid | **Constant** | Constant |
| --- | --- | --- | --- | --- | --- | --- | --- | --- | --- |
|  |  | Relaxed | Strict | Relaxed | Strict | Relaxed | Strict | **Relaxed** | Strict |
| PS | Log(marginal likelihood) | -10022 | -10030 | -10029 | -9973 | -10057 | -10067 | **-9557** | -10064 |
|  | Log(Bayes factor) | 44 | 37 | 38 | 94 | 9 | - | **510** | 2 |
| SS | Log(marginal likelihood) | -10024 | -10030 | -10029 | -9739 | -10058 | -10067 | **-9353** | -10065 |
|  | Log(Bayes factor) | 43 | 36 | 37 | 328 | 9 | - | **713** | 1 |

DENV4

|  |  | **Skyline** | Skyline | Exponential | Exponential | Skygrid | Skygrid | Constant | Constant |
| --- | --- | --- | --- | --- | --- | --- | --- | --- | --- |
|  |  | **Relaxed** | Strict | Relaxed | Strict | Relaxed | Strict | Relaxed | Strict |
| PS | Log(marginal likelihood) | **-9393** | -9411 | -9407 | -9427 | -9412 | -9434 | -9412 | -9434 |
|  | Log(Bayes factor) | **42** | 23 | 27 | 7 | 22 | 0 | 22 | - |
| SS | Log(marginal likelihood) | **-9394** | -9412 | -9408 | -9427 | -9413 | -9435 | -9412 | -9435 |
|  | Log(Bayes factor) | **41** | 23 | 27 | 7 | 22 | 0 | 23 | - |
